# Positive and Negative Emotion Classification Based on Multi-channel

**DOI:** 10.3389/fnbeh.2021.720451

**Published:** 2021-08-26

**Authors:** Fangfang Long, Shanguang Zhao, Xin Wei, Siew-Cheok Ng, Xiaoli Ni, Aiping Chi, Peng Fang, Weigang Zeng, Bokun Wei

**Affiliations:** ^1^Department of Psychology, Nanjing University, Nanjing, China; ^2^Centre for Sport and Exercise Sciences, University of Malaya, Kuala Lumpur, Malaysia; ^3^Institute of Social Psychology, School of Humanities and Social Sciences, Xi’an Jiaotong University, Xi’an, China; ^4^Key & Core Technology Innovation Institute of the Greater Bay Area, Guangdong, China; ^5^Faculty of Engineering, University of Malaya, Kuala Lumpur, Malaysia; ^6^School of Sports, Shaanxi Normal University, Xi’an, China; ^7^Department of the Psychology of Military Medicine, Air Force Medical University, Xi’an, China; ^8^Xi’an Middle School of Shaanxi Province, Xi’an, China

**Keywords:** EEG, emotion classification, support vector machine, decision tree, back propagation neural network, k-nearest neighbor

## Abstract

The EEG features of different emotions were extracted based on multi-channel and forehead channels in this study. The EEG signals of 26 subjects were collected by the emotional video evoked method. The results show that the energy ratio and differential entropy of the frequency band can be used to classify positive and negative emotions effectively, and the best effect can be achieved by using an SVM classifier. When only the forehead and forehead signals are used, the highest classification accuracy can reach 66%. When the data of all channels are used, the highest accuracy of the model can reach 82%. After channel selection, the best model of this study can be obtained. The accuracy is more than 86%.

## Introduction

Emotions play a critical role in everyday life, reflecting a person’s current physical and mental state and significantly impacting cognition, communication, and decision-making. Emotions are generally considered to have two dimensions: arousal and valence, with arousal referring to the emotion’s intensity; valence referring to the specific emotional content, divided into positive, negative, and neutral feelings (Kim et al., 2013; Bailen et al., 2019). Positive emotions can enhance subjective well-being and promote physical and mental health, while persistent negative emotions will affect people’s physical and mental health and work status (Gupta, 2019). Different emotions arise in response to external environmental stimuli and are accompanied by changes in personal representations and psychological reactions, measured and identified by scientific methods (Wolf, 2015).

Previous studies have shown that many signals enable us to identify emotions. The most intuitive expression, voice, posture signals, EEG, ECG, EMG, breathing, and other physiological signals can also measure an emotion. Among many signals that can reflect emotional changes, EEG, with high temporal resolution and non-artifactual characteristics, has been valued by many researchers and is a standard method for emotion recognition. There are some features in EEG signals, which have a strong ability of emotion classification. Petrantonakis and Hadjileontiadis (2010) proposed using higher-order crossover features to extract EEG features, tested four different classifiers, and finally implemented a robust emotion classification method. Chen et al. (2019) verified that the feature of differential entropy significantly affects sentiment classification. In the distribution of brain regions related to emotion classification, there are some differences in the response of different brain regions to different emotions. However, most studies on emotion classification were based on multi-channel EEG signals (Gonzalez et al., 2019; Goshvarpour and Goshvarpour, 2019). Many valuable achievements have been obtained regarding the differences and functions of different brain regions in emotion classification. For example, a study shows that the prefrontal lobe and occipital lobe greatly contribute to emotion classification (Asghar et al., 2019). Firpi and Vogelstein (2011), in a feature selection research work, it is concluded that electrodes F3, F4, T8 have the best effect on emotion classification.

Although there have been many studies on emotion recognition of EEG signals, several problems are still not clear in previous studies. First of all, most of the available data sets for emotion recognition use image, video, audio, and other ways to induce emotional changes. Some researchers used EEG signals from subjects’ happiness, anger, sadness, and joy videos to classify these four types of emotions in related studies (Calix et al., 2012). Other studies classify data based on emotions such as happiness, relaxation, grief, and fear (Alazrai et al., 2018). Therefore, the current criteria for emotion classification are varied due to the high complexity and abstractness of emotion itself. As a result, many researchers fail to reach a unified emotional classification standard when doing work related to emotion recognition. In practice, some studies directly use the label of evoked data as the label of the final emotion classification, but it may exist under the negative emotion but does not induce the negative emotion. Therefore, there is confusion in the sample classification at the beginning. Secondly, in the extraction of EEG signals, the previous research found the most relevant features of emotion from EEG signals. At present, four kinds of features have been used for the emotion classification of EEG signals: time-domain features, frequency domain features, statistical features, and time-frequency domain features (Torres et al., 2020). Although many of the above features have been reported, there is still no detailed research to show which EEG signal feature combinations are most significantly related to emotion classification. Recently, researchers have proposed that differential entropy has a good classification effect in emotion classification. The characteristics of energy proportion and differential entropy have significant effects on the two-channel emotion classification (Al-Nafjan et al., 2017). However, Duan et al. (2013) pointed out that in emotion classification, the degree of discrimination of signals in high-frequency bands is greater than that in low-frequency bands, indicating that differential entropy feature classification in different frequency bands is different still controversial. In addition, previous studies have shown that different brain regions choose to extract features, and finally, there are differences in the effect of emotion classification (Lin et al., 2009; Zhong et al., 2020).

Therefore, a question worth studying is, when we use the differential entropy extracted from different brain regions as the feature of emotion classification, what is the difference in the classification effect? Which brain region will extract the features to achieve the best classification effect? Third, there is a tendency to use as few channels as possible to identify emotions for portability. One study found that when using only the forehead electrode (Fp1 and Fp2) and using the gradient lifting Decision Tree (DT) algorithm to classify happiness and sadness, its accuracy can also reach 95.78% (Al-Nafjan et al., 2017). However, no studies have been conducted to compare the effect of dual and multi-channel classification simultaneously. Is the classification effect of two-channel still better than that of multi-channel under the same experimental setting?

Based on the above problems in emotion recognition research, this study focused on emotions consisting of validity and arousal when classifying emotions and focused on positive and negative emotions within these two categories. The EEG signals were extracted when the subjects watched positive and negative videos, and four classifiers were selected to identify emotion classification. In this study, Support Vector Machine (SVM), DT, Back Propagation Neural Network (BPNN), and k-Nearest Neighbor (kNN) algorithms were used to examine the classification effect of energy share and differential entropy features, the classification effect of differential entropy features in different frequency bands, and the classification effect of differential entropy features in different brain regions. Meanwhile, in the analysis channel, the emotion recognition is carried out on the multi-channel and prefrontal lobe dual-channel data to explore whether there is a difference in the classification accuracy.

## Materials and Methods

### Participants

Twenty-six participants (age range to 18–20, *M* = 19, *SD* = 0.48; 50% female) were recruited through flyers. The subjects had standard visual or corrected visual acuity, normal hearing, and no significant emotional problems or psychiatric disorders by the State-Trait Anxiety Inventory (STAI) and Beck Depression Inventory (BDI). No coffee or alcoholic beverages were consumed within 24 h before the start of the experiment. All subjects signed an informed consent form and received some remuneration at the end of the experiment. If the subjects did not accept the film’s content during the experiment, they could choose not to watch the film or terminate the experiment. The study protocol was approved by the Ethics Committee of the Xian Jiaotong University.

### Stimuli and Procedure

This study used videos that evoked emotion. We used the revised dynamic video library by Deng et al. (2017), which contains eight emotional states: happy, sad, and neutral. Each emotional state consisted of eight video clips, a total of 64 video clips. The length of each video was 60 s. There was no significant difference in invalidity and emotional arousal between videos of the same type. Since this study focused on the binary classification algorithm for positive and negative emotions, four negative segments and four positive segments were selected for the subjects to watch. During the collection, the subjects watched the video of the corresponding emotion theme for 4 min. Each subject was provided with an individual rating in the valence-arousal-dominance-liking four dimensions, ranging from 1 to 9, one being the smallest and nine being the largest. After watching a video, the 9-point rating scale was used to evaluate their subjective emotional experience while watching the video. In this study, emotions were analyzed in terms of two dimensions: valence and arousal. If an individual’s score is greater than 4.5, the arousal/valence level is high; whereas if the individual’s score is less than 4.5, the arousal/valence level is low (Koelstra et al., 2012). The subjects took a 2-min break after the score was completed to help regain their calm.

### EEG Data Acquisition and Preprocessing

The data set was collected in an anechoic darkroom at Xi’an Jiaotong University and significantly reduced noise, reverberation, and electromagnetic interference. Brain electric activity was measured from 32 channels using a modified 10- to 20-system electrode cap (Neuroscan Inc.). All EEGs were continuously sampled at 1,000 Hz. An electrode was placed on the forehead as the ground, and the nose’s tip was the recording reference. EEG was amplified using a 0.1–100 Hz bandpass. The vertical EOG was recorded with electrodes placed above and below the left eye, and the horizontal EOG was recorded with the electrodes placed outboard of both eyes. All electrode impedances were maintained below ten kΩ.

Raw EEG data pre-processing was performed using EEGlab software (Version R2013b, San Diego, USA), an open-source toolbox running MATLAB environment (Version R2013b, MathWorks, USA). Pre-processing of resting EEG data included average reference. Continuous EEG data were band-pass filtered between 0.5 and 45 Hz and a notch filter between 48 and 52 Hz; These segments were then visually inspected to remove those with oculomotor or head movement artifacts. Data were segmented into 2-s–epochs. Eye movement artifacts were corrected using individual independent component analysis (ICA) by removing the corresponding components based on the particular activation curve (Mennes et al., 2010). EEG epochs contaminated by strong muscle artifacts and any EEG epochs with amplitude values exceeding ±80 μV at the electrodes were manually rejected.

#### Multi-channel Sentiment Classification Analysis

**Figure 1:**
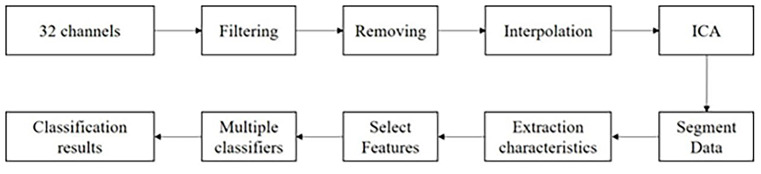
Flow chart of multi-channel emotion classification algorithm.

Based on the conventional EEG data processing scheme, when using multi-channel EEG data for analysis, the data processing and analysis scheme framework in this study is shown in Figure 1.

#### Sample Division

In this study, the emotional state is divided according to the 9-point rating scale, and the EEG clips that do not match the emotional state and video stimulus tags are deleted. After preprocessing, the pure EEG was divided into segments with a length of 1 s. For the whole data set, 5,456 samples were divided, including 2,756 positive emotions and 2,700 negative emotions.

#### Feature Extraction

The EEG signals after pre-processing were sliced into segments of 1 s length. For the whole data set, 5,456 samples can be sliced, including 2,756 positive emotions and 2,700 negative emotions. According to the features used in conventional EEG signal analysis, without considering the specialization to deal with the emotion classification problem, 59-dimensional features were selected for this study, which is a total of 1888-dimensional features for all 32 channels, and this set of features can be used as the baseline features for this study. The 59-dimensional features can be seen in Table 1.

**Table 1:** Basic characteristics of EEG signals.

No.	Features	No.	Features	No.	Features
1	Mean	21	High δ-wave energy	41	β-wave differential entropy
2	Squared difference	22	δ-wave energy	42	γ-wave differential entropy
3	Standard deviation	23	Theta wave energy	43	Total frequency band center of gravity frequency
4	Maximum value	24	Low α wave energy	44	δ-wave center of gravity frequency
5	Minimum value	25	Highαwave energy	45	θ wave center of gravity frequency
6	Median	26	αwave energy	46	α-wave center of gravity frequency
7	Skewness	27	Low β wave energy	47	β-wave center of gravity frequency
8	Kurtosis	28	Highβwave energy	48	γ-wave center of gravity frequency
9	Zero crossover value	29	βwave energy	49	Total frequency band frequency variability
10	Amplitude	30	Low γ wave energy	50	δ-wave frequency variability
11	First-order Difference	31	Highγwave energy	51	Theta wave frequency variability
12	First level difference normalization	32	γ-wave energy	52	α-wave frequency variability
13	Second level difference	33	δ-wave energy share	53	β-wave frequency variability
14	Second level difference normalization	34	θ-wave energy share	54	γ-wave frequency variability
15	Hjorth Mobility	35	α wave energy ratio	55	Sample entropy
16	Hjorth complexity	36	β-wave energy	56	Approximate entropy
17	Fractal dimension	37	γ-wave energy ratio	57	Fuzzy entropy
18	Instability index	38	δ-wave differential entropy	58	Ranking entropy
19	Total energy	39	θ-wave differential entropy	59	Spectral entropy
20	Low δ-wave energy	40	α-wave differential entropy		

It has been shown that the energy share of the sub-band and the differential entropy feature for the emotion classification problem is more effective. The frequency band energy and its percentage can be calculated directly by obtaining the spectrum through the signal’s Fourier transform.

Differential entropy is an extension of Shannon’s entropy, which is defined by the following equation (Shi et al., 2013):

$$h(X)=-\int_{X}f(x)\log\;(f(x))\;dx$$

where X is the EEG signal time series and f(x) is the probability density function of X. If the EEG sequence X obeys a normal distribution, then the differential entropy of the sequence is

$$h(X)=-\int_{X}\frac{1}{\sqrt{2\pi \sigma^{2}}}e^{-\frac{{\left(x-\mu \right)}^{2}}{2\sigma^{2}}}\log\left(\frac{1}{\sqrt{2\pi \sigma^{2}}}e^{-\frac{{\left(x-\mu \right)}^{2}}{2\sigma^{2}}}\right)$$

$$dx=\frac{1}{2}\log\;(2\pi e\sigma^{2})$$

Referring to the observation of Shi et al. (2013) on EEG signals, the EEG signals on commonly used frequency bands obey a normal distribution *N(μ, σ^2^)*. Therefore, for a fixed frequency band *i*, the differential entropy can be calculated by the following equation:

$$h_{i}(X)=\frac{1}{2}\log\;(2\pi e\sigma_{i}^{2})$$

where $\sigma_{i}^{2}$ represents the variance of the EEG sequence *X* on frequency band *i*. And the EEG sequence *X* in any frequency band *i* has no DC component, i.e., the mean value of *X* is 0. At this time, the variance of *X* can be estimated by using the following equation:

$${\hat{\sigma }}_{i}^{2}=\frac{1}{N}\sum_{n\;=\;1}^{N}x_{n}^{2}$$

{*X_n_*} which is the sequence X. Also, by Parseval’s theorem, know that:

$$\sum_{n\;=\;1}^{N}x_{i}^{2}=\frac{1}{N}\sum_{k=1}^{N}{\left|X_{k}\right|}^{2}=P_{i}$$

where {*X_k_*} is the FFT result of {*X_n_*}, *P_i_* representing the energy spectrum on frequency band *i*. From the above derivation, it can be derived that:

$$h_{i}(X)=\frac{1}{2}\log\;(2\pi e\sigma_{i}^{2})=\frac{1}{2}\log\;(P_{i})+\frac{1}{2}\log\;\left(\frac{2\pi e}{N}\right)$$

Since the length of all samples is 1 s, *N* is a constant, and the latter term of the above equation is a constant, which can be disregarded from the classification point of view. Also, the coefficient 1/2 does not affect the performance of the feature in classification.

After obtaining the energy spectrum of each frequency band of the sample, the logarithm can be obtained to represent the differential entropy feature of the EEG signal in that frequency band. This study used this method to calculate the differential entropy and used it as a classification feature.

A gradient boosting DT is chosen to select the most effective channel and features from multi-channel multi-features (GBDT). The algorithm’s feature contribution rate index is used as the basis for feature selection to filter the features that contribute the most to the classification and simplify the model by feature selection to improve the classification efficiency.

#### Classification Algorithm

In this study, four algorithms, Support Vector Machine (SVM), DT, BPNN, and k-Nearest Neighbor (kNN), were chosen to explore the advantages of the happy and sad emotion dichotomous classification problems. When using the SVM algorithm, the kernel function selected in this article is a linear function, and the hyperparameters of the support vector machine are optimized through grid search. When using the BP neural network algorithm, the number of nodes in the input layer is determined according to the number of eigenvalues of each part. The number of nodes in the output layer is set to two, because this article mainly classifies positive and negative emotions. The value of the number of hidden layer nodes is determined by the following formula: $m=\sqrt{n1}$, where n is the number of input layer nodes, l is the number of output layer nodes, and m is the number of hidden layer nodes. When using the DT algorithm, first, the classification model of the DT is generated by learning the training set; second, the model is used to classify samples of unknown types. This study used the C4.5 DT algorithm, and the segmentation index is the information gain rate. When using the knn algorithm, this study used the brute method to select the best k.

After the classifier algorithm is determined, we evaluate the classification performance by dividing the sample data into 10 equal segments that are based on 10-fold cross-validation. Specifically, each round uses nine segments as the training subset and the remaining one segment as the test subset. Thus, the datasets are k non-overlapping data sets, and the datasets are not perfectly consistent with each other. The evaluation metric of correctness is calculated in each trial, and the generalization ability of the model is finally evaluated by averaging the evaluation metrics after k trials. The basic steps of fold cross-validation are as follows:

(a) The original data set is divided into 10 subsets with as balanced sample size as possible;
(b) The first subset is used as the test set, and the second to the ninth subsets are combined as the training set;
(c) Use the training set to train the model and calculate the results of multiple evaluation metrics under the test set;
(d) Repeat steps 2–3, and use subsets two to 10 as the test set in turn;
(e) Calculate the average value of each evaluation index as the final result.

### Dual-Channel EEG Emotion Classification Process

Considering the need for emotion classification in forehead dual-channel portable devices, an algorithmic flow for emotion classification using only forehead electrode signals was also designed in this study, shown in the figure below.

**Figure 2:**
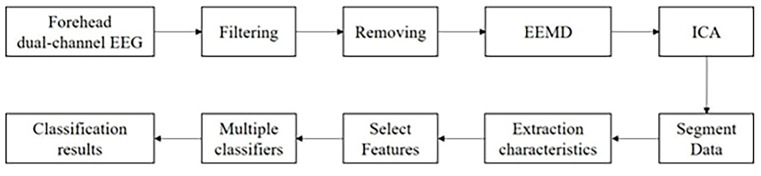
Flow chart of dual-channel emotion classification algorithm.

As shown in Figure 2, there are two changes compared to the multichannel EEG classification process. The number of channels is reduced, and the bad leads cannot be replaced by the peripheral leads’ interpolation of signals. The invalid signals must all be removed entirely in the stage of debugging. The number of channels is too small to apply the ICA algorithm directly, so the Ensemble Empirical Mode Decomposition (EEMD) technique needs to be introduced to decompose each channel’s EEG signal. EEMD is an improved algorithm of EMD that can extract the eigenmode components (IMFs) of the original signal that are not subject to the modal mixing phenomenon (Olesen et al., 2016). After ICA of all IMF components, the EEG can be removed, and the subsequent process is the same as in the case of multiple channels.

## Results

According to the above model training and testing scheme, we arranged five sets of experiments with control variables to find the classification algorithm to achieve the best results gradually.

This study compares each classification model’s strengths and weaknesses using the training set accuracy and the test set accuracy. Also, to take into account the measurement of the generalization ability of the model, two schemes were used on the current data collected from 12 subjects, one is to combine the data from all subjects, train one model and validate its accuracy, and the other is to train the model for each subject separately and validate the accuracy on their respective models, and finally find the average accuracy. In this study, all the subjects’ data were trained and tested together as a whole, and the accuracy rates were calculated separately and averaged as an individual.

### Classification Effects on the Baseline Feature Set

In the baseline feature set of all 1,888 dimensions, the classification effect of each classifier is shown in Table 2.

**Table 2:** Classification effect on the baseline feature set.

Classifier	SVM		DT		BP		kNN	
Category	Training set	Test set	Training set	Test set	Training set	Test set	Training set	Test set
Whole	0.6634	0.6362	0.9880	0.7839	0.8962	0.7949	0.8109	0.7428
Single	0.6995	0.6437	0.9313	0.8127	0.9683	0.9061	0.7996	0.7630

### The Classification Effect of Energy Occupation Ratio and Differential Entropy Feature

A study showed that the energy occupation ratio and differential entropy features have significant effects on two-channel emotion dichotomous classification, so the effects of extending these two sets of features to multiple channels are first verified. Also, to test the effectiveness of linear normalization and standard normalization methods on classification, the effect of different normalized features on these classifiers was added. The results of this set of experiments are shown in Table 3.

**Table 3:** Effect of energy proportion and differential entropy on emotion classification.

Classifier	SVM		DT		BP		kNN	
Category	Training set	Test set	Training set	Test set	Training set	Test set	Training set	Test set
Experiment 2–1	Energy ratio + Differential entropy							
Whole	0.7887	0.7406	0.9765	0.7099	0.8960	0.8079	0.8652	0.8017
Single	0.7772	0.7153	0.9676	0.8219	0.9703	0.9079	0.8641	0.8088
Experiment 2–2	z-score normalization, Energy ratio + Differential entropy							
Whole	0.7638	0.7093	0.9765	0.7099	0.8960	0.8079	0.8317	0.7586
Single	0.7234	0.6590	0.9676	0.8219	0.9703	0.9079	0.8293	0.7601
Experiment 2–3	Linear normalization [0, 1], Energy ratio + Differential entropy							
Whole	0.7673	0.7183	0.9765	0.7099	0.8960	0.8079	0.8451	0.7615
Single	0.7305	0.6641	0.9679	0.8203	0.9666	0.9059	0.8264	0.7673
Experiment 2–4	Differential entropy							
Whole	0.7912	0.7392	0.9773	0.7473	0.9052	0.8292	0.8736	0.8063
Single	0.7757	0.7232	0.9625	0.8179	0.9777	0.9216	0.8513	0.8069
Experiment 2–5	Energy ratio							
Whole	0.6876	0.6353	0.9637	0.5794	0.7556	0.6928	0.7462	0.6956
Single	0.6930	0.6301	0.9558	0.7094	0.8803	0.7953	0.7869	0.7138

From the results of experiments 2–1, it is clear that the group of features, energy percentage, and differential entropy has significantly improved the classification effect over the baseline system, indicating that this group of features can effectively distinguish emotions. Comparing experiments 2–1, 2–2, and 2–3, for this group of features, neither of the two types of normalization can improve the classification effect, so normalization will not be performed in the future when using these two types of features. Experiments 2–4 and 2–5, on the other hand, compared the effect of two features, energy share, and differential entropy, on emotions classification. The results revealed that the energy-occupancy feature’s performance is only comparable to the classification result of the full feature. In contrast, the differential entropy feature’s classification effect is not much different from that of the energy-occupancy + differential entropy feature. Thus, in terms of single metrics, differential entropy is the feature that best captures differences in sentiment.

### Classification Effect of Differential Entropy Features in Different Frequency Bands

Research shows that the difference between the high-frequency band signals is more evident than in the low-frequency band. Therefore, experiments were designed to verify differential entropy features of different frequency bands on emotion classification. The results are shown in the emotion dichotomous classification, the validity of each frequency band EEG signal is ranked as γ > β > δ > α > θ when considered from the perspective of differential entropy features, where γ wave, is the best and β wave is also better. These two frequency bands are influential for the emotion classification problem. The remaining three frequency bands can be considered ineffective in distinguishing emotions compared to the baseline feature set. Comparing experiments 3–1 with 3–6, when β and γ wave features are incorporated simultaneously, it is better than using γ wave features alone. The results of this set of experiments are shown in Table 4.

**Table 4:** The effect of differential entropy in different frequency bands on emotion classification.

Classifier	SVM		DT		BP		kNN	
Category	Training set	Test set	Training set	Test set	Training set	Test set	Training set	Test set
Experiment 3–1	Differential entropy (γ wave)							
Whole	0.8609	0.8219	0.9629	0.7408	0.8416	0.8132	0.8657	0.8308
Single	0.8599	0.8077	0.9597	0.8225	0.9342	0.8950	0.8821	0.8379
Experiment 3–2	Differential entropy (β wave)							
Whole	0.8270	0.7819	0.9589	0.7069	0.8137	0.7867	0.8267	0.7912
Single	0.8245	0.7614	0.9488	0.7856	0.9180	0.8710	0.8523	0.8011
Experiment 3–3	Differential entropy (α wave)							
Whole	0.6894	0.6360	0.9481	0.5915	0.7166	0.6868	0.6941	0.6578
Single	0.6661	0.6024	0.9364	0.6787	0.8175	0.7607	0.7487	0.6816
Experiment 3–4	Differential entropy (θ wave)							
Whole	0.6924	0.6184	0.9461	0.5792	0.7011	0.6620	0.693	0.6131
Single	0.6347	0.5732	0.9357	0.6694	0.8098	0.7480	0.7248	0.6603
Experiment 3–5	Differential entropy (δ wave)							
Whole	0.7315	0.6674	0.9482	0.5931	0.7246	0.6826	0.7429	0.6959
Single	0.6805	0.6067	0.9393	0.6798	0.8284	0.7645	0.7865	0.7186
Experiment 3–6	Differential entropy (β, γ wave)							
Whole	0.8584	0.8226	0.9695	0.7568	0.8771	0.8281	0.8835	0.8422
Single	0.8703	0.8269	0.9566	0.8208	0.9580	0.9095	0.8916	0.8499

### Classification Effects of Differential Entropy Feature for Different Brain Regions

Since γ-wave features are significantly more useful than β-wave features for emotion classification, channel selection was performed using only the differential entropy of γ-wave to determine the best brain regions. The channel selection was performed based on a gradient boosting DT, and Table 5 shows the contribution of 32 channel features to emotion classification. Notably, our view is also supported by the study of Jalilifard et al. (2016), in which the SVM was used to classify emotions using different rhythmic neural oscillations, and it was found that the classification of gamma rhythms was best in the left prefrontal region of the brain (FP1), with β being the second best. Therefore, this part of the results is based mainly on the differential entropy of gamma waves for channel selection.

**Table 5:** The contribution of differential entropy characteristics of each electrode to emotion classification.

Rank	Electrodes	Contribution rate	Rank	Electrodes	Contribution rate	Rank	Electrodes	Contribution rate
1	TP9	0.1383	12	C3	0.0309	23	P7	0.0088
2	Fp2	0.1215	13	P8	0.0197	24	FC1	0.0082
3	T7	0.1013	14	F7	0.0194	25	FT9	0.0082
4	Fp1	0.0758	15	CP6	0.0194	26	Pz	0.0070
5	TP10	0.0682	16	P4	0.0179	27	Cz	0.0065
6	O1	0.0589	17	F3	0.0175	28	F4	0.0049
7	T8	0.0542	18	FC5	0.0173	29	F8	0.0038
8	CP1	0.0387	19	FT10	0.0143	30	P3	0.0037
9	O2	0.0365	20	FC2	0.0124	31	Fz	0.0019
10	Iz	0.0330	21	FC6	0.0103	32	CP2	0.0003
11	C4	0.0320	22	CP5	0.0089			

**Figure 3:**
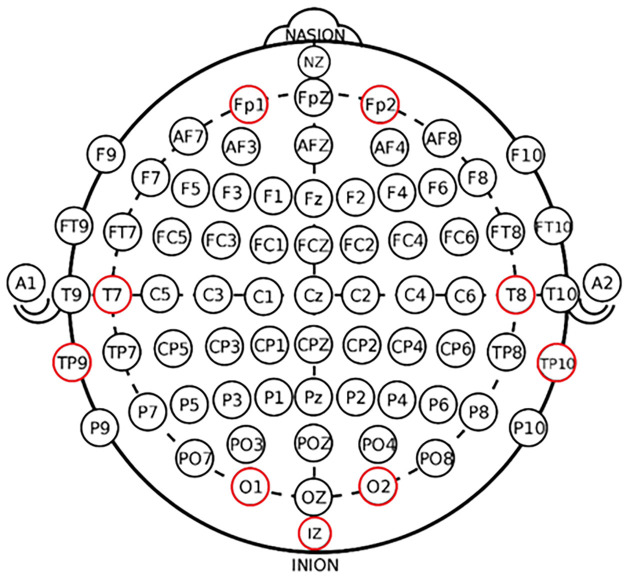
Schematic diagram of the optimal electrode for multi-channel emotion recognition.

Based on each channel’s contribution rate and location distribution, five experiments were designed to verify different feature combinations’ effect on the classification effect. The electrode combinations are shown in Table 6. The experiments were conducted according to the above five sets of electrode combination schemes, and the results are shown in Table 7. From the above experiments, it can be seen that the electrode group corresponding to experiments 5–3 has the best classification effect. Combined with the electrode distribution map, it is clear that the human brain’s lateral ring region is most effective in classifying emotions. The schematic diagram of electrode selection corresponding to experiment 5–3 is shown in Figure 3.

**Table 6:** The contribution of differential entropy characteristics of each electrode to emotion classification.

Experiment		Electrodes
4–1	Forehead	Fp1, Fp2
4–2	Forehead >0.05	Fp1, Fp2, T7, T8, TP9, TP10
4–3	Contribution > 0.03	Fp1, Fp2, T7, T8, TP9, TP10, O1, O2, Iz
4–4	Selection of electrodes along the head loop	Fp1, Fp2, T7, T8, O1, O2
4–5	Only half of the electrode is selected	Fp1, T7, O1

**Table 7:** The effect of differential entropy characteristics under each electrode combination on emotion classification.

Classifier	SVM		DT		BP		kNN	
Category	Training set	Test set	Training set	Test set	Training set	Test set	Training set	Test set
Experiment 5–1	Forehead							
Whole	0.5143	0.5071	0.8970	0.5949	0.6372	0.6296	0.6362	0.6254
Single	0.6703	0.6038	0.9114	0.6956	0.7697	0.7461	0.7642	0.7323
Experiment 5–2	Forehead >0.05							
Whole	0.8591	0.8288	0.9490	0.7460	0.7884	0.7727	0.8551	0.8332
Single	0.8895	0.8455	0.9536	0.8125	0.9046	0.8728	0.8906	0.8588
Experiment 5–3	Contribution > 0.03							
Whole	0.8845	0.8618	0.9591	0.7703	0.8495	0.8332	0.8275	0.8567
Single	0.8970	0.8614	0.9560	0.8275	0.9066	0.8868	0.8924	0.8648
Experiment 5–4	Selection of electrodes along the head loop							
Whole	0.8534	0.8136	0.9465	0.7187	0.8144	0.7933	0.8247	0.7982
Single	0.8598	0.8070	0.9390	0.7703	0.8775	0.8352	0.8473	0.8152
Experiment 5–5	Only half of the electrode is selected							
Whole	0.7881	0.6827	0.9144	0.6428	0.7027	0.6956	0.8082	0.6957
Single	0.7669	0.6814	0.9193	0.7180	0.8041	0.7722	0.7849	0.7596

### Classification Algorithm Selection

Summarizing the above four sets of experiments, the following conclusions can be drawn from the four classification algorithms used in this study:

(1) For DTs and BP neural networks, there is a significant difference, usually about 10%, between the effect of training the models separately for 12 subjects than the effect of training the models together for 12 subjects’ data.
(2) For DTs, the test set’s accuracy is on average more than 20% lower than that of the training set, which shows the severe overfitting of DTs.
(3) SVM and kNN are close to each other, and there is no obvious problem of overfitting and poor generalization performance.
(4) When using SVM for classification, under the premise of channel selection, its accuracy can be as high as 86%, which is better than other classifiers.

### Dual-Channel Dichotomy Effect

After several rounds of experimental comparison, in the two-channel two-classification problem, the best feature combination is the energy proportion and differential entropy of the δ, θ, α, β, and γ bands of the two channels, with a total of 20-dimensional features. The best results are shown in Table 8. As can be seen from the above table, for the dual-channel two-classification problem, the classification effects of using SVM classifier, DT, BP, and kNN are 66%, 60%, 66%, and 64%, respectively. However, considering the pursuit of the model’s generalization ability in the actual classification, the overall performance is taken as the main index. Therefore, under this premise, the best classification model should still choose the SVM classifier; its accuracy can reach 66%.

**Table 8:** The effect of dual-channel 20-dimensional features on emotion classification.

Classifier	SVM		DT		BP		kNN	
Category	Training set	Test set	Training set	Test set	Training set	Test set	Training set	Test set
Whole	0.7270	0.6630	0.9365	0.6037	0.6907	0.6648	0.6894	0.6435
Single	0.7025	0.6227	0.9352	0.6886	0.8100	0.7609	0.7679	0.7035

## Discussion

In this study, we used positive and negative emotion videos to induce emotions in subjects and extracted EEG features of different emotions based on multichannel and prefrontal channels to investigate the effect of classification of positive and negative emotions.

We first obtained the classification results of the four classifiers based on a total of 1,888 features across all channels. In this part of the results, the best performing classifiers are DT and BP neural network, which can achieve an overall correct rate of 78% and 79%, while SVM and kNN only have 63% and 74% correct rates. However, since too many features can cause redundancy of information, in the second section, we use energy share and differential entropy for sentiment classification based on our previous study. Our results show that the difference between the classification effect of “energy share + differential entropy” and that of a single “differential entropy” indicator is not significant, which indicates that differential entropy is the feature that best reflects the difference in sentiment if only a single indicator is considered. The results are consistent with Zheng and Lu (2015); that is, the differential entropy feature is the most stable and outstanding in emotion classification. In the third part of the results report, we focus on the use of differential entropy as a metric to explore the classification effects at different frequency bands. With this part of the analysis, our results show that in terms of the feature classification effect of differential entropy in each frequency band, the order of the validity of EEG signals is γ > β > δ > α > θ, in which the effect of γ wave is the best, and the effect of β wave is also better. The differential entropy of these two bands is effective for emotion classification. The result was consistent with Li et al. (2018) using a hierarchical convolution neural network (HCNN). Their study uses a HCNN to classify positive emotional state, neutral emotional state, and negative emotional state. The differential entropy features from different channels are organized into two-dimensional maps to train HCNN. The results show that there is a good classification ability on Beta and Gamma waves. In the fourth part of the result report, based on the previous results, we select only the differential entropy features of the γ-band to analyze the classification accuracy of different brain regions, and the results show that when electrodes are selected along the head loop, there are good results under all four classifiers, and, from the results of the test set, the SVM classifier has the best classification results (86.18% accuracy overall), while the DT, BP, and kNN root class effects were 77.03%, 83.32%, and 85.67%, respectively. This is one point where this study goes beyond previous research in that it identifies which regions have the greatest impact on emotion classification, given the identified extracted features.

The study also compares the feature classification effects of differential entropy in different brain regions. The results showed that when using the differential entropy of γ wave for channel selection, the lateral annular region of the human brain is the most effective for emotion classification. This is a point that this study surpasses previous studies; that is, on the premise of determining the extraction of features, we point out which regions have the greatest impact on emotion classification. Another concern of our study is whether there is a difference in the classification effect between multi-channel and dual-channel. The discussion of this problem is not simply to repeat the experimental process on multiple channels but to select the best feature combination by comparing several groups of experiments. Our results show that the energy ratio and differential entropy of the δ, θ, α, β, and γ bands of the two channels have a good classification effect. When using SVM and BP neural network classifiers, they can reach the classification accuracy of 66%. However, considering the better generalization ability of SVM in previous studies (Yao et al., 2019), we suggest that SVM is the best classifier for the binary classification of positive and negative emotions based on two-channel data. It’s worth noting that, the classification efficiency of multi-channel feature classification is better than two-channel feature classification regardless of the feature classification effect due to the fact that multi-channel has more information and can better represent the information (Lin et al., 2009; Garg and Verma, 2020). Therefore, to pursue higher classification accuracy, as many channels as possible should be selected to explore emotion classification.

Finally, the classification effects of the four classifiers were compared. In multi-channel EEG emotion classification, SVM and kNN are better than DT and BP neural network in classification effect and generalization ability. However, the KNN algorithm needs to store many training samples and is not easy to implement on embedded devices. Meanwhile, the computational complexity of prediction increases linearly with the increase of training samples. The SVM algorithm, on the other hand, has stable computational complexity, and the training can be done offline. Therefore, from the perspective of practical applications, SVM is superior to kNN. In the two-channel study, considering the model’s generalization ability, the SVM algorithm is more appropriate. The conclusion of this study once again demonstrates the effectiveness of the SVM algorithm in emotion classification, which is consistent with the research of Nie et al. (2011). In their research, we use the multi-feature fusion and SVM classifier method to study the second classification of emotion, and the accuracy is 87.53%. Altogether, this study extends the previous findings. Based on the multi-channel emotion-evoked EEG data, the two types of emotions can be effectively classified using the energy proportion and differential entropy of frequency bands best effect by using an SVM classifier. When only the signals of the two channels of the forehead are used, the highest classification accuracy can reach 66%. When the data of all channels are used, the highest accuracy of the model can reach 82%. After channel selection, the best model can be obtained in which the accuracy can reach 86%. The electrodes in the optimal channel combination scheme are all located in the lateral annular region of the human brain, which provides a theoretical basis for the follow-up development of portable headband emotion monitoring equipment.

## Limitations

This study also has some limitations. First, the current study uses the subject-dependent way for emotion recognition, even if a single subject’s data is used to train the emotion classifier for the subject. When the subject is changed, it is necessary to train a new classifier for the subject. There are great defects in generalization ability. Second, although our research shows that better emotion classification results can be achieved by using an SVM classifier, some studies have shown that when using a deep learning model to classify emotions, its accuracy is 3.54% higher than that of the traditional SVM algorithm (Zheng et al., 2014), which suggests that future research can focus on deep learning model to explore further how to achieve more efficient emotion classification results.

## Data Availability Statement

The raw data supporting the conclusions of this article will be made available by the authors, without undue reservation.

## Ethics Statement

The studies involving human participants were reviewed and approved by the Ethics Committee of the Xian Jiaotong University. The patients/participants provided their written informed consent to participate in this study.

## Author Contributions

XW and FL conceived the study and designed the study. SZ, S-CN, and XN collected the data. AC, PF, WZ, and BW analyzed the data and was involved in writing the manuscript. All authors contributed to the article and approved the submitted version.

## Conflict of Interest

The authors declare that the research was conducted in the absence of any commercial or financial relationships that could be construed as a potential conflict of interest.

## Publisher’s Note

All claims expressed in this article are solely those of the authors and do not necessarily represent those of their affiliated organizations, or those of the publisher, the editors and the reviewers. Any product that may be evaluated in this article, or claim that may be made by its manufacturer, is not guaranteed or endorsed by the publisher.
